# PP73 Integrating Valid Recommendations In The Clinical Practice Guidelines Updating Process Using GRADE Methodology

**DOI:** 10.1017/S0266462324002423

**Published:** 2025-01-07

**Authors:** Trinidad Sabalete-Moya, Juan Antonio Blasco-Amaro, Francisco Javier Gracia-San Román, Ruth Engelhardt-Pintiado

## Abstract

**Introduction:**

The development of clinical practical guidelines (CPGs) has been evolving towards a standard methodology based on GRADE. While reviewing CPGs, we found valid recommendations and no evidence to update. Including these initial recommendations in addition to new recommendations as part of the scope can improve understanding of the issue.

**Methods:**

To develop a standardized approach to the writing of recommendations when updating a CPG, we consulted several methodological handbooks: Scottish Intercollegiate Guidelines Network (SIGN) (2011 and 2019 version), National Institute for Health and Care Excellence (NICE) last update (2024), New Zealand Guidelines Group (NZGG) grading system, and Oxford Centre for Evidence-Based Medicine (OCEBM) Levels of Evidence 2. We compared the wording and specific symbols used to express strength of recommendations and quality of evidence with those used by GRADE, as described in the methodology handbook for developing clinical practice guidelines in the Spanish National Health System (SNS).

**Results:**

A table was developed to express the equivalence of wording and symbols of quality of evidence and grading of recommendations to align the expression of SIGN, NICE, OCEBM, and NZGG methods with GRADE terminology. SIGN and NICE methodologies did not utilize specific symbols to express strength of recommendations; SIGN did not utilize the GRADE approach to assess the quality of evidence. The NZGG system applied its own classification for quality assessment and grading of recommendations. Oxford OCEBM keeps the terminology of level of evidence and grading of recommendations.

**Conclusions:**

Using wording and symbols equivalence to standardize the recommendations of different methods to GRADE could assist the process of updating CPGs with the GRADE-Adolopment approach. Recommendations that are still valid and have been developed rigorously could be integrated in an updated CPG avoiding the duplication of efforts, but this generates uncertainty, especially when the quality assessment is focused on the studies designs.

